# Expression of BAFF and BAFF-R in Follicular Lymphoma: Correlation with Clinicopathologic Characteristics and Survival Outcomes

**DOI:** 10.1371/journal.pone.0050936

**Published:** 2012-12-13

**Authors:** Ya-Jun Li, Wen-Qi Jiang, Hui-Lan Rao, Jia-Jia Huang, Yi Xia, Hui-Qiang Huang, Tong-Yu Lin, Zhong-Jun Xia, Su Li, Zhi-Ming Li

**Affiliations:** 1 State Key Laboratory of Oncology in South China, Guangzhou, People's Republic of China; 2 Department of Medical Oncology, Sun Yat-Sen University Cancer Center, Guangzhou, People's Republic of China; 3 Department of Pathology, Sun Yat-Sen University Cancer Center, Guangzhou, People's Republic of China; 4 Department of Hematological Oncology, Sun Yat-Sen University Cancer Center, Guangzhou, People's Republic of China; University of Barcelona, Spain

## Abstract

**Background:**

B-cell activation factor (BAFF) and BAFF-receptor (BAFF-R) play crucial roles in the viability and proliferation of malignant lymphoma cells. Limited information exists regarding expression profiles and the prognostic role of BAFF and BAFF-R in follicular lymphoma (FL). We sought to determine the expression profiles of BAFF and BAFF-R in FL and to evaluate the correlation of BAFF and BAFF-R expression with clinicopathologic characteristics and outcome of FL. Correlation between expression levels of BAFF detected by immunohistochemical (IHC) and serum levels of BAFF was also evaluated.

**Methods:**

Paraffin-embedded specimens from 115 patients were immunohistochemically examined for BAFF and BAFF-R expression. Expression levels were dichotomized into low versus high categories based on immunostaining intensity. The correlation of BAFF and BAFF-R expression with clinicopathologic characteristics and patient outcome was assessed. Serum levels of BAFF in 35 of the 115 patients with IHC data were measured by Enzyme-linked Immunosorbent assay (ELISA).

**Results:**

BAFF and BAFF-R were expressed in 88.7% (102/115) and 87.8% (101/115) of the cases, respectively. BAFF expression was significantly correlated with only one clinicopathologic feature: Ann Arbor stage. No significant correlation was found between expression levels of BAFF detected by IHC and serum levels of BAFF detected by ELISA. High expression of BAFF-R, but not BAFF, was significantly correlated with inferior progression-free survival (PFS; *P* = 0.013) and overall survival (OS; *P* = 0.03). High expression of BAFF-R, bulky disease, and elevated lactate dehydrogenase were correlated with inferior PFS and OS in multivariate analysis. A prognostic scoring system incorporating these 3 risk factors identified 3 distinct prognostic groups with 5-year PFS of 59.4%, 41.9%, and 10.7% and OS of 91.3%, 79.7%, and 45.8%, respectively.

**Conclusions:**

Most patients with FL variably express BAFF and BAFF-R. High expression of BAFF-R, but not BAFF, may be an independent risk factor for PFS and OS in FL.

## Introduction

Follicular lymphoma (FL) is the second most common lymphoma in western countries, accounting for 22% of newly diagnosed cases [1]. FL also is the most frequent indolent lymphoma both in western and Asian countries [2]. Although several prognostic indexes have been established to stratify patients into risk groups, including the widely accepted Follicular Lymphoma International Prognostic Index (FLIPI) and the more recently developed Follicular Lymphoma International Prognostic Index 2 (FLIPI2) [3], [4], their value is based on a limited number of clinical and laboratory prognostic factors. The prognostic value of immunohistochemical markers expressed in FL remains to be determined.

B-cell activation factor (BAFF; also known as B-lymphocyte stimulator [BLyS]) existing either on the cell surface as a type 2 transmembrane protein or in the serum as a soluble form of this transmembrane protein after plasma membrane cleavage, is a member of tumor necrosis factor (TNF) family [5], [6]. BAFF plays critical roles in B-cell homeostasis, viability, and malignant transformation by binding and activating three receptors: BAFF receptor (BAFF-R; also known as BR3), transmembrane activator and calcium modulator and cyclophilin ligand interactor (TACI), and B-cell maturation antigen (BCMA) [5]–[8]. Numerous studies have indicated that interaction between BAFF and BAFF-R and BCMA promotes malignant B-cell survival and proliferation through both autocrine and paracrine pathways *in vitro* [9]–[11]. Reports by Kern et al. and Fu et al. have demonstrated that BAFF-R protected B-cell non-Hodgkin's lymphoma (NHL-B) against apoptosis and activated NF-κB pathways in the plasma membrane [10], [11]. In addition, BAFF-R can also promote NHL-B survival and proliferation by functioning as a transcriptional regulator by binding to IKKβ and histone H3 in the nucleus [10]. In contrast to the role of BAFF-R, several studies have suggested that TACI was a negative regulator of B-cell activation, and TACI-deficient mice showed fatal lymphoproliferation and autoimmune diseases [12]–[14]. Furthermore, increasing evidence has demonstrated that serum BAFF levels both in non-Hodgkin's lymphoma (NHL) and Hodgkin's lymphoma patients are elevated compared with those of healthy controls, and patient outcome is poorer when serum BAFF levels are higher [15]–[18]. However, the expression level of BAFF in tumor specimens from lymphoma remains unclear. In addition, although several previous immunohistochemical studies have confirmed that BAFF-R is variably expressed in FL [15], [19]–[22], the correlation between expression levels and clinicopathologic features of disease and patient outcome remains to be elucidated.

This study was therefore conducted to determine the distributions and patterns of expression of BAFF and BAFF-R in FL. Additionally, another aim was to evaluate the correlation of levels of BAFF and BAFF-R expression with clinicopathologic characteristics of disease and outcome in FL.

## Materials and Methods

### Ethics Statement

This study was approved by the Institutional Review Board of the National Cancer Institute, as well as ethics committees of Sun Yat-Sen University Cancer Center. The study was performed in accordance with the Declaration of Helsinki and the institutional guidelines of the local ethics committee. All patients provided their written informed consent for their blood samples and other medical information to be stored in our hospital database, and we obtained separate consent for use of research.

### Patient selection

Newly diagnosed patients were eligible for this study if they had diagnostic biopsy specimens showing follicular lymphoma. After review and reclassification according to the WHO classification [23], 115 patients with FL treated in a single institution (Sun Yat-Sen University Cancer Center) were included between July 1998 and September 2009. Adequate clinical information and follow-up data were available for all patients.

### Immunohistochemistry

Immunohistochemical (IHC) analysis was performed using two monoclonal antibodies: anti-BAFF antibody (Buffy 2, ab16081, 1∶400, Abcam) and anti-BAFF receptor antibody (11C1, ab16232, 1∶400, Abcam). The anti-BAFF antibody can recognize both membrane-bound and soluble BAFF protein. Sections (4 µm thick) were cut from each formalin-fixed paraffin block, deparaffinized, and incubated at 121°C in citrate buffer (pH 6.0) for 10 min for antigen retrieval. A routine immunohistochemistry method was performed for immunostaining the above antigens, as described previously [22]. Based on the methods of Hans et al. [24] and Wada et al. [22], staining was defined as positive for BAFF when the protein was detected in ≥30% of tumor cells with membrane and/or cytoplasm staining, and for BAFF-R when the protein was detected in ≥30% of tumor cells with membrane staining, respectively. The immunohistochemical results for BAFF and BAFF-R were classified as follows: −, no staining; 1+, weak staining; 2+, moderate staining; 3+, strong staining. Negative immunochemical results were defined as <30% positive tumor cells, regardless of staining intensity. Based on the staining intensity of BAFF and BAFF-R, all patients were divided into two groups: a BAFF or BAFF-R low expression group (patients with − or 1+ staining) versus BAFF or BAFF-R high expression group (patients with 2+ or 3+ staining). The immunostaining was evaluated by 2 experienced hematopathologists who were blinded to the clinicopathologic features and outcome of patients. Discordance between these two pathologists were discussed and decided at consensus conferences.

### BAFF ELISA

Serum BAFF levels were determined by Enzyme-linked Immunosorbent assay (ELISA) kits using a mouse monoclonal antibody against BAFF (Quantikine Human BAFF Immunoassay; R&D Systems, Minneapolis, MN, USA). All blood samples were obtained at diagnosis and then centrifuged at 4°C. Serum was collected and quickly frozen at –80°C until assay. A routine ELISA assay method was performed according to the manufacturer's protocol and as described previously [18]. The minimal detectable dose of BAFF was 2.68pg/ml. All samples were analyzed in duplicates and each value was calculated as the mean ± standard deviation (SD) of duplicate samples.

### Response criteria and statistical methods

Treatment response was assessed in accordance with the International Working Group Recommendations for Response Criteria for non-Hodgkin's lymphoma [25]. Progression-free survival (PFS) was defined as the interval between the date of diagnosis and the date of first relapse, progression, death, or last follow-up. Overall survival (OS) was defined from the day of diagnosis until the time of death or last follow-up. Correlations between expression levels of BAFF or BAFF-R and clinicopathologic factors, FLIPI, and FLIPI2 index were analyzed by chi-square tests. Correlation between expression levels of BAFF detected by IHC and serum levels of BAFF was assessed using Spearman's rank correlation test. The log-rank test and Kaplan-Meier method were used for univariate survival analysis. Multivariate analysis was performed according to the Cox proportional hazard model. A two-tailed *P*-value≤0.05 was considered to be statistically significant. The statistical software package SPSS 16.0 (SPSS, USA) was used for statistical calculations.

## Results

### Clinicopathologic characteristics of the patients at diagnosis

The main clinicopathologic characteristics of the patients are summarized in Table 1. Totally, 115 patients with FL (66 males [57.4%]) were included in this study. The median age at diagnosis was 49 years (range, 14–74 years), and 20.9% patients were 60 years or older. FLIPI (n = 115) and FLIPI2 (n = 85) were retrospectively assessed. The distribution of FLIPI scores indicated that 44.3, 27.0, and 28.7% of patients, respectively, were at low (score, 0–1), intermediate (score, 2), and high (score, ≥3) risk. The distribution of FLIPI2 scores indicated that 64.7, 21.2, and 14.1% of patients, respectively, were at low (score, 0–1), intermediate (score, 2), and high (score, ≥3) risk. Initial treatments included combination chemotherapy+rituximab ± involved-field radiotherapy (IFRT) (n = 41, 13 cases received IFRT), chemotherapy ± IFRT (n = 58, 17 cases received IFRT), single agent rituximab therapy (n = 9), and others (interferon [n = 1], IFRT alone [n = 3], surgery alone [n = 2], watchful waiting [n = 1]). The regimens of chemotherapy in the initial treatment included: CHOP (cyclophosphamide, doxorubicin, vincristine, and prednisone) or CHOP-like (n = 77), CVP (cyclophosphamide, vincristine, and prednisone) (n = 5), FND (fludarabine, mitoxantrone, dexamethasone) (n = 15), single agent chlorambucil (n = 1). The second-line and subsequent therapy usually included the salvage therapy used for diffuse large B-cell lymphoma according to the National Comprehensive Cancer Network (NCCN) guideline. The first-line and subsequent treatment details are listed in Table 2. Although the proportion of patients ever received rituximab during the course of their disease was significant higher in the high-BAFF-R expression group than in the low-BAFF-R expression group, no significant difference was found in the other treatment modalities of patients according to low- versus high-BAFF and BAFF-R expression groups. Sixty-nine cases received rituximab, 43 cases received radiotherapy, and 6 cases received high-dose therapy with autologous stem cell transplantation during the course of their diseases. There were 39 deaths (33.9%) during a median follow-up of 4.6 years (range, 0.7–12.7 years). In the surviving patients, the median follow-up time was 5.9 years (range, 2.5–12.7 years). The median overall survival for all 115 cases was 10.6 years. The 5-year PFS and OS rates for all 115 patients were 38% and 73.8%, respectively. The 10-year PFS and OS rates for all patients were 34.5% and 56.8%, respectively.

**Table 1:** Clinicopathologic characteristics for 115 patients with FL.

Characteristics	No.	%
**Age**		
Median (range, years)	49 (14–74)	
**Gender**		
Male	66	57.4
Female	49	42.6
**Histologic grade**		
1	31	27.0
2	27	23.5
3	57	49.5
**Growth pattern**		
F	72	62.6
FAD	37	32.2
D	6	5.2
**BCL-2 status (n = 112)**		
Positive	101	90.2
Negative	11	9.8
**ECOG PS**		
<2	105	91.3
≥2	10	8.7
**B symptoms**		
Yes	31	27.0
No	84	73.0
**Bone marrow involvement**		
Yes	12	10.4
No	103	89.6
**Bulky disease (≥7 cm)**		
Yes	29	25.2
No	86	74.8
**LDH**		
Normal	89	77.4
Elevated	26	22.6
**Ann Arbor stage**		
I/II	47	40.9
III/IV	68	59.1
**FLIPI**		
Low risk (0–1)	51	44.3
Intermediate risk (2)	31	27.0
High risk (≥3)	33	28.7

FL, follicular lymphoma; F, follicular; FAD, follicular and diffuse; D, diffuse; ECOG PS, Easter Cooperative Oncology Group performance status; LDH, lactate dehydrogenase; FLIPI, Follicular Lymphoma International Prognostic Index.

**Table 2:** First-line and subsequent therapy in patients with FL.

	BAFF expression			BAFF-R expression		
Treatment	Low, n (%)	High, n (%)	*P*	Low, n (%)	High, n (%)	*P*
**Rituximab in first line**						
Yes	22 (37.9)	28(49.1)	0.226	17 (38.6)	33 (46.5)	0.410
No	36 (62.1)	29 (50.1)		27 (61.4)	38 (53.5)	
**Ever received rituximab**						
Yes	31 (53.4)	38 (66.7)	0.148	21 (47.7)	48 (67.6)	0.034
No	27 (46.6)	19 (33.3)		23 (52.3)	23 (32.4)	
**Anthracyclines in first line**						
Yes	47 (81.0)	45 (78.9)	0.780	35 (79.5)	57 (80.3)	0.924
No	11 (19.0)	12 (21.1)		9 (20.5)	14 (19.7)	
**Ever received anthracyclines**						
Yes	51 (87.9)	50 (87.7)	0.972	37 (84.1)	64 (90.1)	0.335
No	7 (12.1)	7 (12.3)		7 (15.9)	7 (9.9)	
**Therapy in first line**						
Rituximab-chemotherapy	17 (29.3)	24 (42.1)	0.176	14 (31.8)	27 (38.0)	0.743
Chemotherapy	30 (51.7)	28 (49.1)		25 (56.8)	33 (46.5)	
Rituximab alone	5 (8.6)	4 (7.0)		3 (6.8)	6 (8.4)	
Other therapy*	6 (10.3)	1 (1.8)		2 (4.5)	5 (7.0)	
**IFRT in first line**						
Yes	20 (34.5)	13 (22.8)	0.166	10 (22.7)	23 (32.4)	0.265
No	38 (65.5)	44 (77.2)		34 (77.3)	48 (67.6)	

FL, follicular lymphoma; BAFF, B-cell activation factor; BAFF-R, B-cell activation factor receptor;

* Initial therapy of the other 7 cases were as follows: one case received interferon treatment; 3 cases only received local radiotherapy as initial treatment; 2 cases only received surgery treatment; one case received watchful waiting as initial management; IFRT, involved-field radiotherapy.

The main pathology data are summarized in Table 1. Thirty-one (27%) FL cases were histologic grade 1; 27 (23.5%), grade 2; and 57 (49.6%), grade 3. Seventy-two (62.6%) of the cases had a follicular pattern of growth, 37 (32.2%) had a mixed follicular and diffuse pattern of growth, and 6 (5.2%) had a diffuse pattern of growth. Of 112 evaluable cases, 101 (90.2%) were positive for BCL-2.

### Distributions and patterns of expression of BAFF and BAFF-R in FL

**Figure 1:**
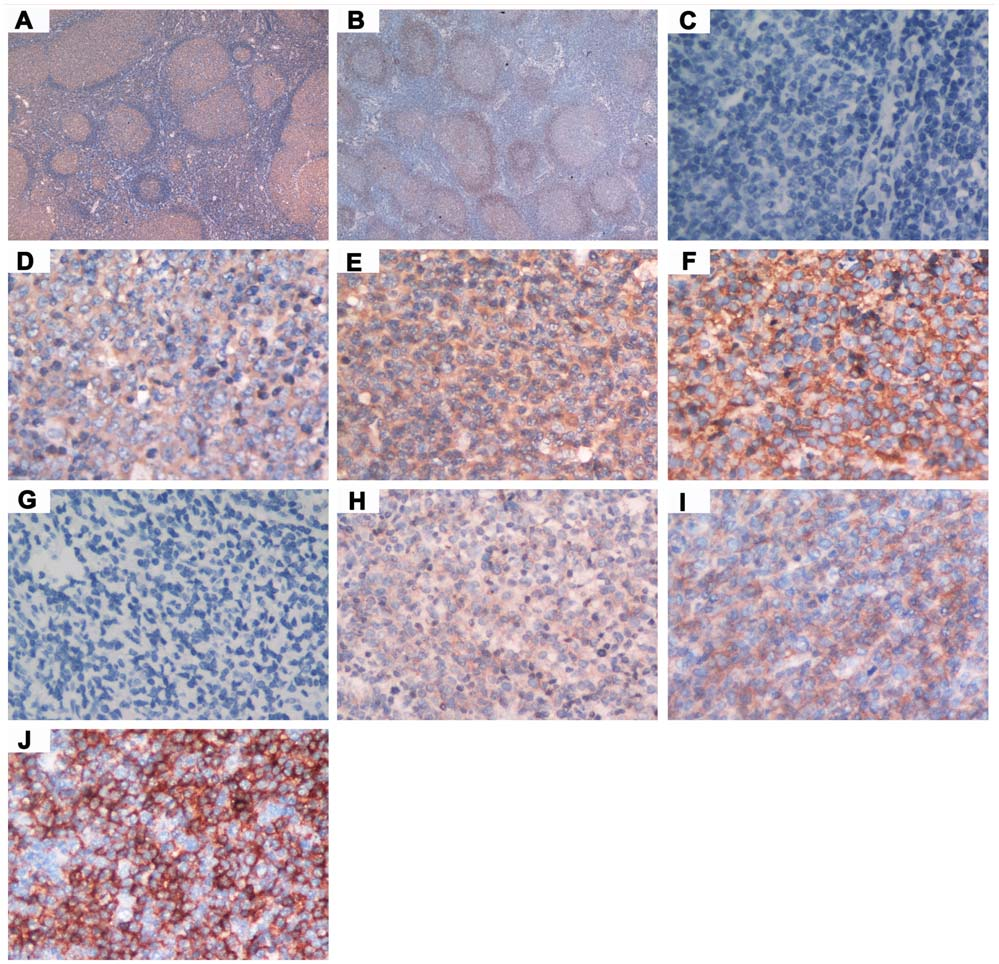
Typical distributions of BAFF and BAFF-R expression in follicular lymphoma (FL) and representative cases with different immunostaining intensity for BAFF and BAFF-R. (A) Distribution of BAFF expression in tumor specimen of FL. (B) Distribution of BAFF-R expression in tumor specimen of FL. (C) Negative staining (−) for BAFF. (D) Weak staining (1+) for BAFF. (E) Moderate staining (2+) for BAFF. (F) Strong staining (3+) for BAFF. (G) Negative staining (−) for BAFF-R. (H) Weak staining (1+) for BAFF-R. (I) Moderate staining (2+) for BAFF-R. (J) Strong staining (3+) for BAFF-R.

In FL with typical follicular or follicular and diffuse growth pattern, BAFF was mainly expressed in the germinal center (GC) areas and only faintly and infrequently in the mantle zone and interfollicular compartment (Figure 1A). In contrast to BAFF, BAFF-R was predominantly and strongly expressed in the mantle zone, often but weakly expressed on GC tumor cells, and often absent in the interfollicular areas (Figure 1B). The vast majority of patients were variably positive for BAFF (102/115, 88.7%) and BAFF-R (101/115, 87.8%). Staining was weak to moderate in most BAFF-positive patients: weak (n = 44; 43.1%), moderate (n = 50; 49.0%), and strong (n = 8; 7.8%). Nevertheless, among BAFF-R-positive cases, staining was weak, moderate, and strong, respectively, in 30 (29.7%), 32 (31.7%), and 39 (38.6%) cases. Representative cases illustrating different staining intensity are shown in Figure 1. The rates of low and high expression of BAFF were 50.4% (58/115) and 49.6% (57/115); and the rates of low and high expression of BAFF-R were 38.3% (44/115) and 61.7% (71/115), respectively. The detailed expression profiles of BAFF and BAFF-R according to pathological features are listed in Table 3. The expression levels of BAFF and BAFF-R (low group vs. high group) were not related to the main histopathologic characteristics, as shown in Table 3.

**Table 3:** Distribution of BAFF and BAFF-R expression in 115 patients with FL according to histopathologic features.

	BAFF expression					BAFF-R expression				
	Low, n (%)		High, n (%)		*P* *	Low, n (%)		High, n (%)		*P* *
Feature	−	1+	2+	3+		−	1+	2+	3+	
**Grade of FL**										
1	4 (12.9)	13 (41.9)	12 (38.7)	2 (6.5)		1 (3.2)	7 (22.6)	11 (35.5)	12 (38.7)	
2	6 (22.2)	9 (33.3)	12 (44.5)	0 (0)		8 (29.6)	5 (18.5)	5 (18.5)	9 (33.3)	
3	3 (5.3)	22 (38.6)	26 (45.6)	6 (10.5)	0.478	5 (8.8)	18 (31.6)	16 (28.1)	18 (31.6)	0.196
**Growth pattern**										
F	8 (11.1)	29 (40.3)	31 (43.1)	4 (5.6)		8 (11.1)	15 (20.8)	25 (34.7)	24 (33.3)	
FAD	3 (8.1)	14 (37.8)	16 (43.2)	4 (10.8)		4 (10.8)	13 (35.1)	6 (16.2)	14 (37.8)	
D	2 (33.3)	1 (16.7)	3 (50.0)	0 (0)	0.865	2 (33.3)	2 (33.3)	1 (16.7)	1 (16.7)	0.123
**BCL-2 (n = 112)**										
Positive	12 (11.9)	39 (38.6)	43 (42.6)	7 (6.9)		10 (9.9)	27 (26.7)	28 (27.7)	36 (35.6)	
Negative	0 (0)	4 (36.4)	6 (54.5)	1 (9.1)	0.373	3 (27.3)	2 (18.2)	3 (27.7)	3 (27.7)	0.806

BAFF, B-cell activation factor; BAFF-R, B-cell activation factor receptor; FL, follicular lymphoma; F, follicular; FAD, follicular and diffuse; D, diffuse;

* The *P*-values were calculated with Chi-square test for comparison of low and high expression of BAFF or BAFF-R according to different histopathologic features.

### Relationship between the expression of BAFF and BAFF-R, clinical features, and survival outcomes

When divided into low- versus high-BAFF and BAFF-R expression groups according to immunostaining intensity, no relationship of BAFF and BAFF-R was found to most of main clinical features. However, high expression of BAFF but not BAFF-R was significantly more frequent in patients with Ann Arbor stage III/IV diseases than those with stage I/II diseases (*P* = 0.045, Table 4).

**Table 4:** Correlation between main clinical features of 115 patients with FL and expression of BAFF and BAFF-R.

	BAFF expression			BAFF-R expression		
Features	Low, n (%)	High, n (%)	*P*	Low, n (%)	High, n (%)	*P*
**Age, years**						
<60	44 (48.4)	47 (51.6)		37 (40.7)	54 (59.3)	
≥60	14 (58.3)	10 (41.7)	0.384	7 (29.2)	17 (70.8)	0.303
**Gender**						
Male	33 (50.0)	33 (50.0)		22 (33.3)	44 (66.7)	
Female	25 (51.0)	24 (49.0)	0.914	22 (44.9)	27 (55.1)	0.207
**ECOG PS**						
<2	55 (52.4)	50 (47.6)		42 (40.0)	63 (60.0)	
≥2	3 (30.0)	7 (70.0)	0.307	2 (20.0)	8 (80.0)	0.367
**LDH**						
Normal	47 (52.8)	42 (47.2)		32 (36.0)	57 (64.0)	
Elevated	11 (42.3)	15 (57.7)	0.346	12 (46.2)	14 (53.8)	0.347
**Hemoglobin (g/dL)**						
≥12	48 (52.7)	43 (47.3)		36 (39.6)	55 (60.4)	
<12	10 (41.7)	14 (58.3)	0.334	8 (33.3)	16 (66.7)	0.577
**β2-MG (n = 85)**						
Normal	30 (51.7)	28 (48.3)		24 (41.4)	34 (58.6)	
Elevated	10 (37.0)	17 (63.0)	0.207	6 (22.2)	21 (77.8)	0.085
**Ann Arbor stage**						
I/II	29 (61.7)	18 (38.3)		18 (38.3)	29 (61.7)	
III/IV	29 (42.6)	39 (57.4)	0.045	26 (38.2)	42 (61.8)	0.995
**FLIPI**						
Low risk	28 (54.9)	23 (45.1)		23 (45.1)	28 (54.9)	
Intermediate risk	16 (51.6)	15 (48.4)		9 (29.0)	22 (71.0)	
High risks	14 (42.4)	19 (57.6)	0.530	12 (36.4)	21 (63.6)	0.337

FL, follicular lymphoma; BAFF, B-cell activation factor; BAFF-R, B-cell activation factor receptor; ECOG PS, Easter Cooperative Oncology Group performance status; LDH, lactate dehydrogenase; β2-MG, β2-microglobulin; FLIPI, Follicular Lymphoma International Prognostic Index.

**Figure 2:**
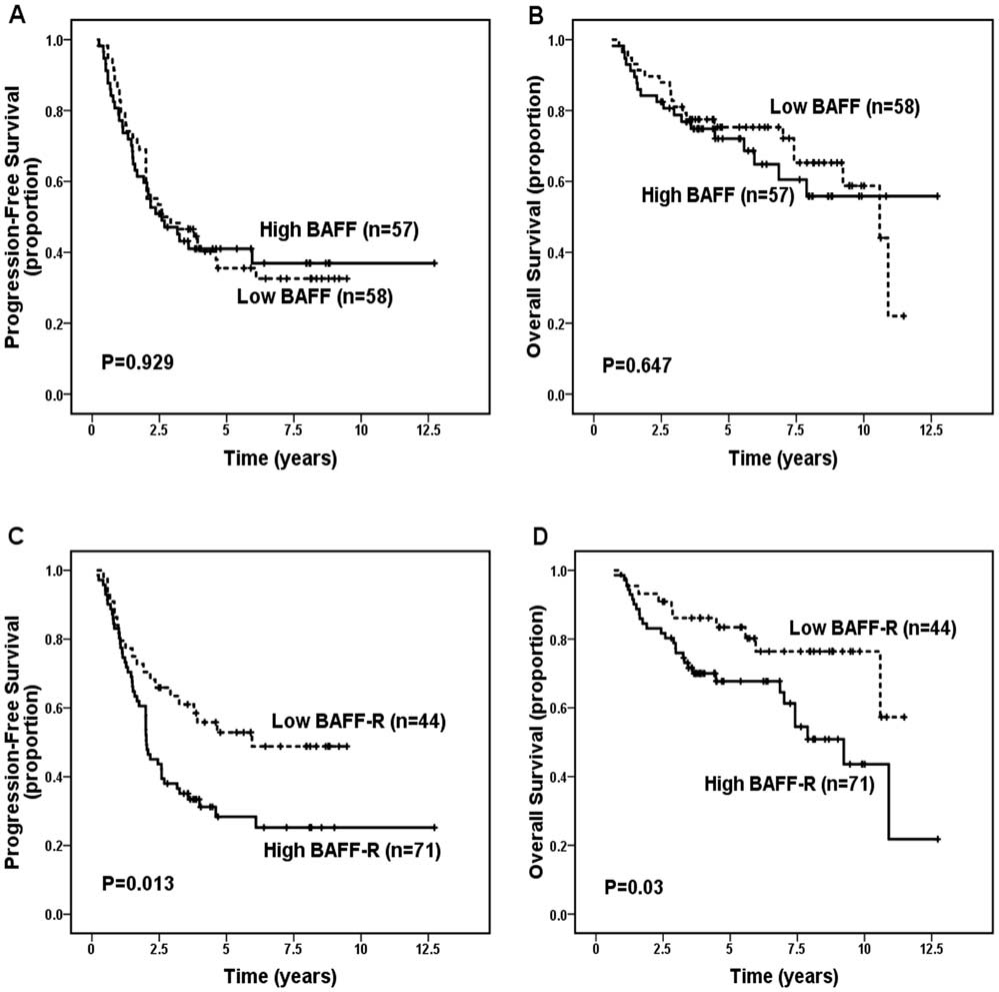
Progression-free survival (PFS) and overall survival (OS) for 115 patients with follicular lymphoma according to BAFF and BAFF-R expression levels. (A) No significant difference in PFS was noted between low- and high-BAFF expression groups (*P* = 0.929). (B) No significant difference in OS was noted between low- and high-BAFF expression groups (*P* = 0.647). (C) Patients with high-BAFF-R expression showed significant inferior PFS (*P* = 0.013). (D) Patients with high-BAFF-R expression showed significant inferior OS (*P* = 0.03).

**Figure 3:**
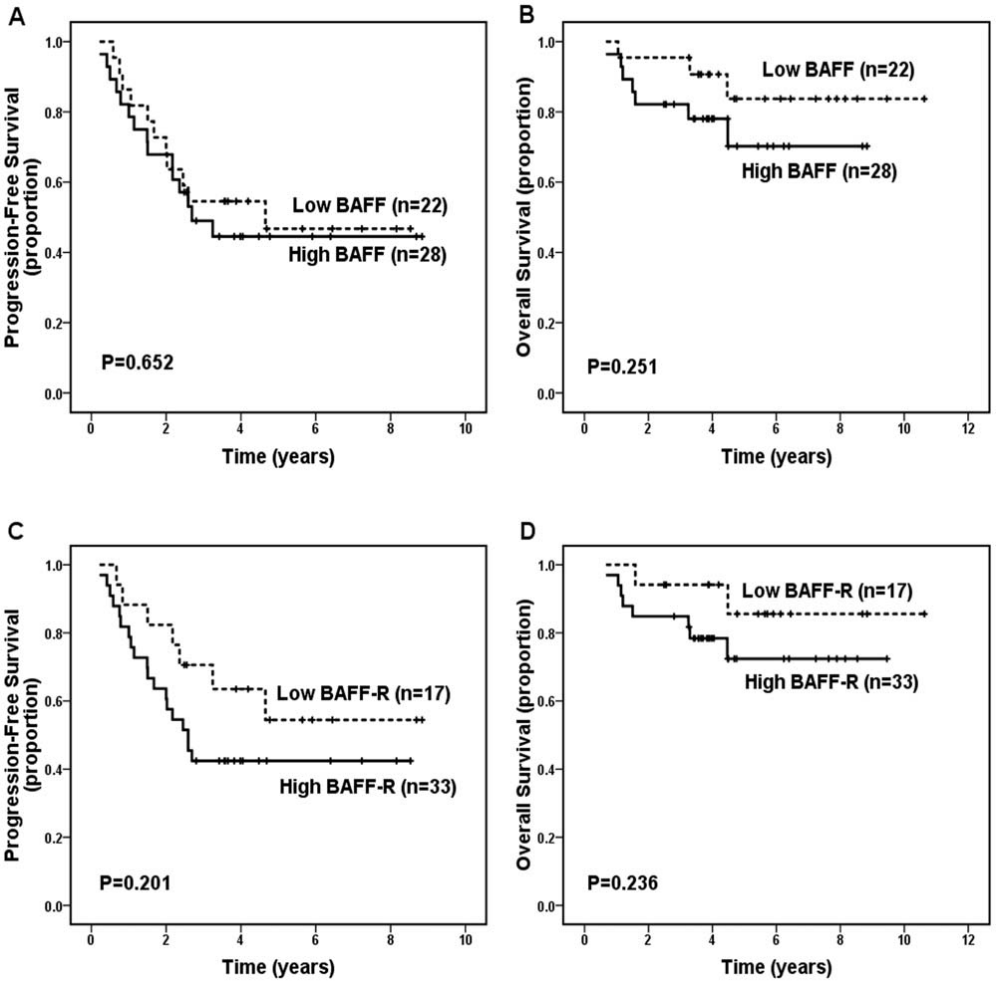
Progression-free survival (PFS) and overall survival (OS) for 50 patients with follicular lymphoma treated with rituximab ± chemotherapy according to BAFF and BAFF-R expression levels. (A) No significant difference in PFS was noted between low- and high-BAFF expression groups (*P* = 0.652). (B) No significant difference in OS was noted between low- and high-BAFF expression groups (*P* = 0.251). (C) No significant difference in PFS was noted between low- and high-BAFF-R expression groups (*P* = 0.201). (D) No significant difference in OS was noted between low- and high-BAFF-R expression groups (*P* = 0.236).

**Figure 4:**
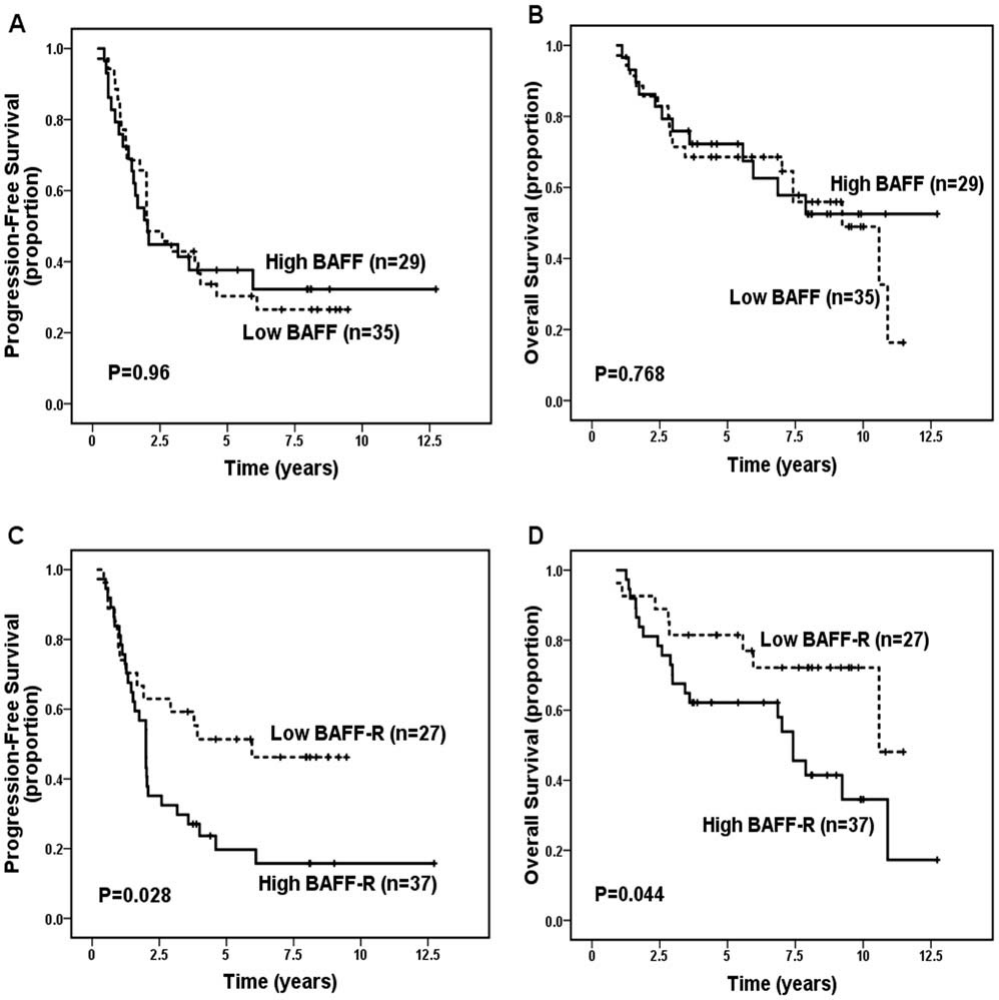
Progression-free survival (PFS) and overall survival (OS) for 64 patients with follicular lymphoma treated with non-rituximab regimens according to BAFF and BAFF-R expression levels. (A) No significant difference in PFS was noted between low- and high-BAFF expression groups (*P* = 0.96). (B) No significant difference in OS was noted between low- and high-BAFF expression groups (*P* = 0.768). (C) Patients with high-BAFF-R expression showed significant inferior PFS (*P* = 0.028). (D) Patients with high-BAFF-R expression showed significant inferior OS (*P* = 0.044).

When patients were dichotomized into negative- versus positive- BAFF and BAFF-R groups, both BAFF and BAFF-R were not significantly correlated with PFS (*P* = 0.398, and *P* = 0.122, respectively) and OS (*P* = 0.202 and *P* = 0.061, respectively). When dichotomized into low- versus high- categories of protein expression, high expression of BAFF was still not significantly correlated with worse PFS (*P* = 0.929, Figure 2A) and OS (*P* = 0.647, Figure 2B), but high expression of BAFF-R was significantly correlated with inferior PFS (*P* = 0.013, Figure 2C) and OS (*P* = 0.03, Figure 2D). Patients with low BAFF-R expression had significantly better 5-year PFS (52.9% vs. 28.4%; *P* = 0.013) and 5-year OS (83.4% vs. 67.7%; *P* = 0.03), and had significant better 10-year PFS (48.8% vs. 25.2%) and OS (76.4% vs. 43.6%). Analysis of all 114 cases receiving treatment after diagnosis (one group treated with rituximab ± chemotherapy [n = 50] and one group treated with non-rituximab regimens [chemotherapy {n = 58}, radiotherapy {n = 3}, surgery {n = 2}, or interferon {n = 1}] [n = 64]) detected only a slight tendency (without statistical significance) toward association of high BAFF and BAFF-R expression with inferior PFS and OS in patients treated with rituximab ± chemotherapy (all *P*>0.05, Figure 3A–3D), however it detected a significant association of high expression of BAFF-R, but not of BAFF, with decreased PFS (*P* = 0.028) and OS (*P* = 0.044) in patients treated with non-rituximab regimens (Figure 4A–4D). In addition, patients with both high BAFF and high BAFF-R showed significant worse OS than those with both low BAFF and BAFF-R (*P* = 0.019).

### Serum BAFF levels

Serum samples were available for 35 of 115 patients with data of IHC. We analyzed the data of serum BAFF levels of these 35 patients. The median level of serum BAFF was 835.78 (range: 414.15–6928.58) pg/ml, and the mean ± SD level was 1,115.50±1,135.01 pg/ml. No significant correlation between expression levels of BAFF detected by IHC and serum levels of BAFF detected by ELISA was found by Spearman's rank correlation test (correlation coefficient = 0.164, *P* = 0.346). The median value (835.78 pg/ml) of BAFF was used as a cut-off point to dichotomize patients. Thus, high BAFF group was defined as ≥835.78 pg/ml (n = 18) while low BAFF group was defined as <835.78 pg/ml (n = 17). There were no significant differences in PFS and OS between high and low BAFF groups (*P* = 0.239 and *P* = 0.573, respectively).

### Univariate analysis for PFS and OS

In univariate analysis among 16 possible unfavorable prognostic factors, 10 factors were significantly associated with poor PFS: B symptoms, Ann Arbor stage III/IV, bone marrow involvement, number of nodal sites ≥5, bulky disease, elevated β2-microglobulin (β2-MG), elevated lactate dehydrogenase, increasing FLIPI, increasing FLIPI2, and high expression of BAFF-R; whereas, 9 factors were significantly associated with poor OS: B symptoms, number of nodal sites ≥5, bulky disease, elevated hemoglobin, elevated β2-MG, elevated LDH, increasing FLIPI, increasing FLIPI2, and high expression of BAFF-R (Table 5). Factors significant at *P*≤0.05 in univariate analysis were included in multivariate analysis except for β2-MG and FLIPI2 (which were excluded because of missing data).

**Table 5:** Prognostic value of risk factors for 115 patients with FL in univariate analysis.

	PFS			OS		
Risk factor	HR	95% CI	*P* ^*^	HR	95% CI	*P* ^*^
Age (y), ≥60 vs. <60	1.24	0.711–2.463	0.445	1.82	0.918–3.597	0.086
Gender, Male vs. female	1.28	0.888–2.053	0.309	1.53	0.793–2.959	0.204
Grade of FL, 3 vs. 1/2	1.04	0.808–1.289	0.868	1.24	0.895–1.707	0.198
Growth pattern, FAD/D vs. F	1.26	0.887–2.024	0.347	1.61	0.844–3.058	0.149
BCL-2 (n = 112), Positive vs. negative	2.27	0.824–6.211	0.113	1.01	0.306–3.323	0.988
B symptoms, Yes vs. no	1.73	1.053–2.855	0.030	2.01	1.054–3.831	0.034
Ann Arbor stage, III/IV vs. I/II	2.08	1.25–3.436	0.005	1.88	0.934–3.802	0.077
BM involvement, Yes vs. no	3.44	1.783–6.623	<0.001	1.29	0.484–3.226	0.646
No. of nodal sites, ≥5 vs. 0–4	2.36	1.467–3.756	<0.001	2.14	1.125–4.051	0.020
Bulky disease (≥7 cm), Yes vs. no	2.35	1.431–3.846	0.001	2.12	1.107–4.082	0.023
Hemoglobin (g/dL), <12 vs. ≥12	1.54	0.902–2.638	0.113	2.19	1.126–4.252	0.021
β2-MG (n = 85), Elevated vs. normal	1.45	1.123–1.874	0.004	5.68	2.609–12.368	<0.001
LDH, Elevated vs. normal	1.78	1.060–2.990	0.029	4.22	2.181–8.157	<0.001
FLIPI, H vs. I vs. L risk	1.75	1.324–2.303	<0.001	2.33	1554–3.482	<0.001
FLIPI2 (n = 85), H vs. I vs. L risk	1.96	1.371–2.791	<0.001	2.77	1.770–4.314	<0.001
BAFF-R expression, High vs. low	1.90	1.135–3.174	0.013	2.19	1.060–4.509	0.030

FL, follicular lymphoma; PFS, progression-free survival; OS, overall survival; HR, hazard ratio; 95% CI, 95% confidence interval; F, follicular; FAD, follicular and diffuse; D, diffuse; BM, bone marrow; β2-MG, β2-microglobulin; LDH, lactate dehydrogenase; FLIPI, Follicular Lymphoma International Prognostic Index; H, high; I, intermediate; L, low; BAFF-R, B-cell activation factor receptor; *P* ^*^≤0.05 for inclusion and retention in multivariate analysis (except for β2-MG and FLIPI2 because of their missing data).

### Multivariate analysis for PFS and OS

**Figure 5:**
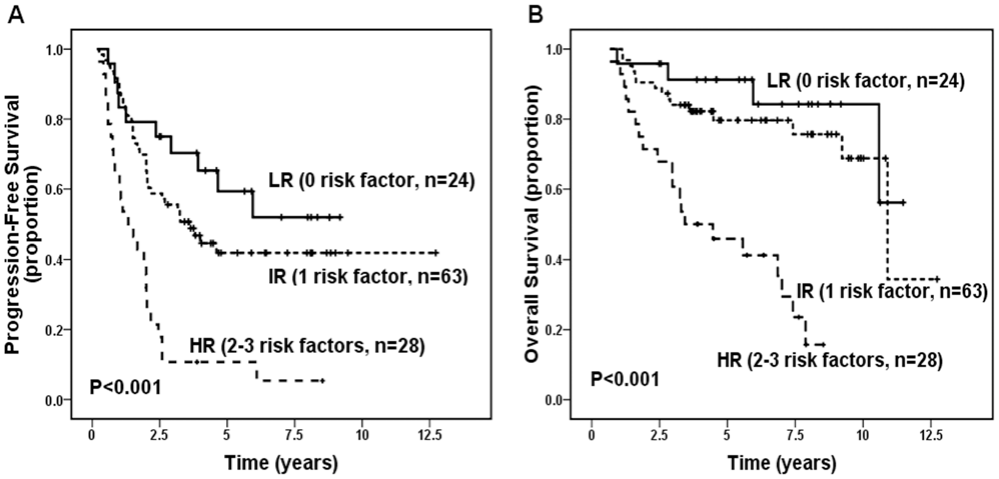
Progression-free survival (PFS) and Overall survival (OS) for all 115 patients with follicular lymphoma according to the prognostic scoring system incorporating the 3 independent risk factors for both PFS and OS. (A) Increasing scores correlated with inferior PFS (*P*<0.001). (B) Increasing scores correlated with inferior OS (*P*<0.001). LR, low risk group (patients with no risk factor); IR, intermediate risk group (patients with 1 risk factor); HR, high risk group (patients with 2–3 risk factors).

Multivariate analysis identified 5 significant independent prognostic factors for shorter PFS (bone marrow involvement [*P* = 0.03], number of nodal sites ≥5 [*P* = 0.020], bulky disease [*P* = 0.001], elevated LDH [*P* = 0.027], and high expression of BAFF-R [*P* = 0.016]) and 3 significant independent prognostic factors for worse OS (bulky disease [*P* = 0.035], elevated LDH [*P*<0.001], and high expression of BAFF-R [*P* = 0.010]; Table 6). Finally, a prognostic scoring system incorporating these 3 independent risk factors for both PFS and OS was devised and used to stratify our 115 patients into a low-risk (0 risk factor), intermediate-risk (1 risk factors), and high-risk (2–3 risk factors) groups. On Kaplan-Meier analysis, the 3 risk groups showed clear separation into 3 survival groups (PFS, *P*<0.001; OS, *P*<0.001, Figure 5A, 5B). Patients in the low- (n = 24), intermediate- (n = 63), and high- (n = 28) risk groups had a 5-year PFS of 59.4%, 41.9%, and 10.7% and OS of 91.3%, 79.7%, and 45.8%, respectively, and 10-year PFS of 51.9%, 41.9%, and 5.4% and OS of 84.2%, 68.8% and 15.7%, respectively. For patients who ever received rituximab (n = 69), the new prognostic model also could differentiate different patients with different outcomes (5-year OS of low-, intermediate-, and high-risk group was 100%, 81.5%, and 56.8%, respectively, 10-year OS was 100%, 81.5% and 26.5%, respectively, both *P* = 0.001). Similarly, the FLIPI also had significant prognostic value for OS in both all 115 patients and patients ever used rituximab (*P* = 0.001 and *P*<0.001, respectively).

**Table 6:** Prognostic value of risk factors for 115 patients with FL in multivariate analysis.

	PFS			OS		
Risk factor	HR	95% CI	*P*	HR	95% CI	*P*
B symptoms, Yes vs. no	–	–	0.398	–	–	0.284
Ann Arbor stage, III/IV vs. I/II	–	–	0.584	–	–	–
BM involvement, Yes vs. no	2.25	1.082–4.655	0.030	–	–	–
No. of nodal sites, ≥5 vs. 0–4	1.86	1.101–3.135	0.020	–	–	0.334
Bulky disease (≥7 cm), Yes vs. no	2.32	1.379–3.886	0.001	2.05	1.052–3.984	0.035
Hemoglobin (g/dL), <12 vs. ≥12	–	–	–	–	–	0.520
LDH, Elevated vs. normal	1.85	1.073–3.185	0.027	4.56	2.335–8.907	<0.001
FLIPI, H vs. I vs. L risk	–	–	0.997	–	–	0.119
BAFF-R expression, High vs. low	1.92	1.127–3.257	0.016	2.61	1.259–5.425	0.010

FL, follicular lymphoma; PFS, Progression-free survival; OS, Overall survival; HR, hazard ratio; 95% CI, 95% confidence interval; −, not applicable; BM, bone marrow; LDH, lactate dehydrogenase; FLIPI, Follicular Lymphoma International Prognostic Index; H, high; I, intermediate; L, low; BAFF-R, B-cell activation factor receptor.

## Discussion

Although a growing body of literature has demonstrated that BAFF and BAFF-R proteins are closely associated with the clinicopathologic features of lymphomas, most of these studies are mainly concerned with the impact of elevated serum BAFF on patient outcome [15]–[18], [22]. The expression of BAFF and BAFF-R in tumor specimens from patients with FL and the prognostic role of these two proteins in patient outcome have not been completely elucidated. This study was therefore performed to examine the expression profiles of BAFF and BAFF-R in FL tumor specimens and to evaluate the correlation of expression levels of BAFF and BAFF-R with clinicopathologic characteristics and outcome of FL.

Reports indicate that numerous tumor cell types express BAFF, including B cell chronic lymphocytic leukemia (B-CLL) [15], [26], diffuse large B-cell lymphoma (DLBCL) [15], FL [15], mantle cell lymphoma [15], and pre-B acute lymphoblastic leukemia [27]. However, expression patterns in tumor specimens of FL are unclear. In the present study, BAFF was expressed in most cases of FL. This result is in line with previously published data by Novak et al. [15]. However, in contrast to the data of Novak et al. indicating that BAFF expression levels correlate with disease severity [15], our data show no significant variation in levels of BAFF expression with tumor grade and growth pattern and no association of levels of BAFF-R expression with histologic grade and growth pattern, even though the vast majority of patients with FL were BAFF-R positive. These findings are consistent with the findings of several previous studies showing high positive rates of BAFF-R in FL [19], [20], [22], but inconsistent with the findings of Paterson et al. indicating that only 51.4% patients with FL were positive for BAFF-R, and patients with grade 3 FL were often negative for BAFF-R [21]. These discrepancies may be related to patient selection criteria and the limited sample size of some series, but also to variability in technical aspects such as antibodies, different scoring methods, criteria, and cut points.

Increasing evidence shows that elevated serum BAFF levels correlate with poor outcome in patients with Hodgkin's lymphoma [16], [17] and DLBCL [15], [18]. However, in our study, unexpectedly, the serum BAFF level failed to show prognostic significance for survival outcomes in patients with FL. This is partly due to the small number of patients with data of serum BAFF concentrations and obvious difference in biological behavior between DLBCL (aggressive) and FL (indolent). Furthermore, level of BAFF expression in tumor specimens also had no prognostic significance for PFS and OS in patients with FL. Similarly, in another study, we found no correlation of overexpression of BAFF with poor outcome in patients with DLBCL (unpublished data). There are three possible explanations for this. First, since BAFF exists in two forms (soluble and membrane-bound), the anti-BAFF antibody used in our study was specific for both forms. The difference in biological function between the two forms remains unclear. Furthermore, whether the two forms of BAFF have antagonistic biological functions is also unknown. Second, given the findings that TACI mediates negative regulation by binding to BAFF in B cells [12]–[14] and BAFF-R promotes malignant B-cell survival and proliferation by binding to BAFF ligand [9], [10], [15], it is conceivable that BAFF binding to BAFF-R and BAFF binding to TACI are competitive. Third, given that there was no significant correlation between high expression of BAFF detected by IHC and high serum BAFF levels, the results of IHC may only reflect the levels of BAFF expressed in local tumor tissues rather than the overall serum BAFF levels of the host. More investigations are needed to further evaluate the prognostic value of BAFF levels in serum and local tumor specimens in patients with FL.

In our study, regardless of the initial treatment, high expression of BAFF-R was found to be an independent prognostic risk factor for decreased PFS and OS in all 115 patients with FL. Unfortunately, when differences in initial treatment were taken into account, BAFF-R was shown to have no prognostic significance in patients treated with rituximab-containing regimens. The reasons for this remain unclear. Possibly, the addition of rituximab to chemotherapy regimens eliminates the unfavorable prognostic impact of BAFF-R on patient outcome. The lack of prognostic significance may also be partly due to the small number of patients treated with rituximab-containing regimens (n = 50) in this study. Cases more uniformly treated with rituximab are needed to accurately evaluate the prognostic value of BAFF-R in patients with FL.

In multivariate analysis, high expression of BAFF-R was a risk factor for PFS and OS in our patients with FL. This is inconsistent with a previous study by Wada et al. indicating that BAFF-R expression is a favorable prognostic factor for OS in patients with DLBCL [22]. The obvious difference in biological behavior between DLBCL (aggressive) and FL (indolent) may account for this. Further studies are warranted to explore the different impacts of BAFF-R on the prognosis of aggressive and indolent lymphomas.

Although the biological mechanisms underlying the association between high BAFF-R expression and poor prognosis are still incompletely defined, there are two potential explanations to account for this. First, the activation of NF-κB pathway is one of the major mechanisms. After interaction with BAFF-R and other receptors, BAFF activates NF-κB, which leads to the resistance to apoptosis of lymphoma B cells by upregulation of the antiapoptotic proteins Bcl-2, Bcl-xL, and Mcl-1 and downregulation of proapoptotic proteins such as Bax [9], [28], [29], [30], [31]. In addition, the *in vitro* exposure of NHL-B cells to exogenous BAFF showed a reduction in apoptosis and prolonged cell survival [9]. Second, BAFF-R promotes cell proliferation and survival by interaction with IKKβ and NF-κB/c-Rel in the nucleus of neoplastic B-lymphoid cells [10]. Recent study by Fu et al. demonstrates that in addition to activating NF-κB pathways in the plasma membrane, BAFF-R also promotes B-cell NHL survival and proliferation by functioning as a transcriptional regulator through a chromatin remodeling mechanism and NF-κB association [10]. Since there is no significant association between high BAFF expression and high BAFF-R expression in the present study (data not shown), when BAFF expression is low, the latter may be the primary mechanism of BAFF-R promoting neoplastic cells survival and proliferation in FL. However, further studies are warranted to elucidate this issue.

Finally, we devised a prognostic scoring system incorporating high expression of BAFF-R, bulky disease, and elevated LDH (3 independent risk factors for PFS and OS), which allows separation of patients with FL into 3 distinct survival groups. Survival outcome is excellent in patients without any of the 3 risk factors and significantly worse in those with all 3 risk factors. Identifying this high-risk patient population with our prognostic scoring system can be used to aid clinicians in selecting patients best suited for early aggressive therapy. More importantly, similar to FLIPI, our novel model was highly predictive for outcomes also in patients treated with rituximab. However, given the small number of patients treated with rituximab in present study, further external validation cohort would be needed to verify the prognostic value of the present new prognostic scoring system in the era of rituximab.

In conclusion, our study shows that the vast majority of patients with FL are variably positive for BAFF and BAFF-R. Level of BAFF expression correlates with Ann Arbor stage but not with any of the other main clinicopathologic features of FL. BAFF-R, but not BAFF, is an independent prognostic factor for PFS and OS in patients with FL. Additionally, given the ubiquitous expressions of these two proteins and the unfavorable impact of BAFF-R on patient outcome, BAFF and BAFF-R might be potentially important therapeutic targets in FL.
